# *Sox* transcription in sarcosine utilization is controlled by Sigma^54^ and SoxR in *Bacillus thuringiensis* HD73

**DOI:** 10.1038/srep29141

**Published:** 2016-07-12

**Authors:** Qi Peng, Chunxia Liu, Bo Wang, Min Yang, Jianbo Wu, Jie Zhang, Fuping Song

**Affiliations:** 1 State Key Laboratory for Biology of Plant Diseases and Insect Pests, Institute of Plant Protection, Chinese Academy of Agricultural Sciences, Beijing, China; 2 College of Life Sciences, Northeast Agriculture University, Harbin, China

## Abstract

Sarcosine oxidase catalyzes the oxidative demethylation of sarcosine to yield glycine, formaldehyde, and hydrogen peroxide. In this study, we analyzed the transcription and regulation of the *sox* locus, including the sarcosine oxidase-encoding genes in *Bacillus thuringiensis* (Bt). RT-PCR analysis revealed that the *sox* locus forms two opposing transcriptional units: *soxB* (*soxB/E/F/G/H/I*) and *soxR* (*soxR/C/D/A*). The typical −12/−24 consensus sequence was located 15 bp and 12 bp from the transcriptional start site (TSS) of *soxB* and *soxC*, respectively. Promoter-*lacZ* fusion assays showed that the *soxB* promoter is controlled by the Sigma^54^ factor and is activated by the Sigma^54^-dependent transcriptional regulator SoxR. SoxR also inhibits its own expression. Expression from the P*soxCR* promoter, which is responsible for the transcription of *soxC*, *soxD,* and *soxA*, is Sigma^54^-dependent and requires SoxR. An 11-bp inverted repeat sequence was identified as SoxR binding site upstream of the *soxB* TSS. Purified SoxR specifically bound a DNA fragment containing this region. Mutation or deletion of this sequence abolished the transcriptional activities of *soxB* and *soxC*. Thus, SoxR binds to the same sequence to activate the transcription of *soxB* and *soxC*. Sarcosine utilization was abolished in *soxB* and *soxR* mutants, suggesting that the *sox* locus is essential for sarcosine utilization.

Sarcosine is reportedly a potential oncometabolite where in prostate cancer sarcosine may serve as a possible sensitive tumor biomarker through its role in tumor progression and metastasis^1^. Sarcosine oxidase (SOX, EC 1.5.3.1) catalyzes the oxidative demethylation of sarcosine to yield glycine, formaldehyde, and hydrogen peroxide, which as the nitrogen source for bacteria growth^2^. Sarcosine oxidases exist in monomeric, heterodimeric, and heterotetrameric (alpha, beta, gamma, and delta) forms^3^ and mediate creatine and glycine betaine metabolism in bacteria. In *Arthrobacter* sp. 1-IN and *Arthrobacter globiformis*, the *glyA*, *soxBDAG*, and *purU* genes of the sarcosine oxidase operon and the *dmg* gene of the dimethylglycine oxidase operon work together to catabolize glycine betaine to produce serine^4^. In *Arthrobacter* sp. TE1826, the *soxA* gene, which encodes monomeric sarcosine oxidase (MSOX), forms a cluster with the upstream regulator gene *soxR* and the downstream *crnA* and *creA* genes, which encode creatininase and creatinase, respectively, and this Sigma^70^ factor-regulated cluster participates in the metabolism of creatine to glycine^5^. The three enzymes encoded by this operon have been used for the diagnostic assessment of serum creatinine levels^6^. Other studies have shown that the *soxR* gene product SoxR, a member of the LysR family of regulatory proteins, is a negative regulator of *soxA*^7^. A similar genetic organization was observed in *Bacillus* sp. B-0618, where the gene encoding creatinase is located near the gene encoding MSOX^8^. In *Corynebacterium* sp. U-96 and *Corynebacterium* sp. P-1, the genetic organization of *sox* and its nearby genes is similar, which both includes *glyA*, *soxBDAG*, and *purU*. However, the *sdh* gene separates *soxG* and *purU* in *Corynebacterium* sp. U-96. The different organization of the *sox* locus in these species reflects the utilization of sarcosine in different metabolic pathways, e.g., in the metabolism of sarcosine to pyruvate in *Corynebacterium* sp. U-96 and to serine in *Corynebacterium* sp. P-1^9^,^10^.

The *Bacillus cereus* group of gram-positive, spore-forming bacteria, includes *Bacillus cereus*, the causative agent of food poisoning in humans; *Bacillus thuringiensis* (Bt), a pathogenic agent in insects; and *Bacillus anthracis*, the etiological agent of anthrax in mammals^11^,^12^. The organization of the sarcosine oxidase gene and nearby genes is similar in these groups^12^,^13^. A gene encoding a Sigma^54^-dependent transcriptional regulator separates the genes encoding the sarcosine oxidase units in reverse orientation, suggesting that the regulation of the sarcosine oxidase locus in the *B. cereus* group, which occurs through the Sigma^54^ factor, is different from the well-studied regulatory mechanisms of other bacteria.

The alternative Sigma factors are the promoter-recognition subunits of bacterial RNA polymerase holoenzymes^14^. Structural and functional studies have shown these factors can be divided into Sigma^70^ and Sigma^54^ classes. Sigma^54^ promoters have common features: (i) they are devoid of the typical −10/−35 sequences recognized by the Sigma^70^ factor^15^ and have strongly conserved −12/−24 regions^16^; and (ii) they require a positive regulator to stimulate isomerization of the closed complexes of RNA polymerase and the promoter to the corresponding open complexes^17^,^18^. Sigma^54^ plays an important role in the regulation of many metabolic pathways in bacteria^19^,^20^,^21^. The *sigL* gene in *Bacillus subtilis* encodes Sigma^54^ 22,23, and was used to identify five Sigma^54^-dependent transcriptional regulators (or enhancer-binding proteins, EBPs), including LevR, RocR, AcoR, BkdR, and YplP, which regulate the levanase operon, arginine metabolic pathway, acetoin catabolic pathway, branched-chain fatty acid synthetic pathway, and the cold shock response, respectively. However, only two metabolic pathways are known to be controlled by Sigma^54^: the γ-aminobutyric acid (GABA)^24^ and l-lysine metabolism^25^ pathways in Bt HD73. Little is known about other metabolic pathways controlled by Sigma^54^ in the *B. cereus* group.

In this study, we focused on the organization and regulation of the sarcosine oxidase gene and nearby genes (*HD73_3147*-*HD73_3138*) in *B. thuringiensis* subsp. *kurstaki* strain HD73 (Bt HD73)^26^. The *HD73_3147*-*HD73_3138* genes of the *sox* locus were separately designated as *soxI*, *soxH*, *soxG*, *soxF*, *soxE*, *soxB*, *soxR*, *soxC*, *soxD*, and *soxA*. Three SoxR-regulated promoters were identified in the *sox* locus, two of which are Sigma^54^-dependent. The results of this study will provide new insight into the metabolic pathways controlled by Sigma^54^.

## Results

### Characterization of transcription units in the *sox* locus

**Figure 1:**
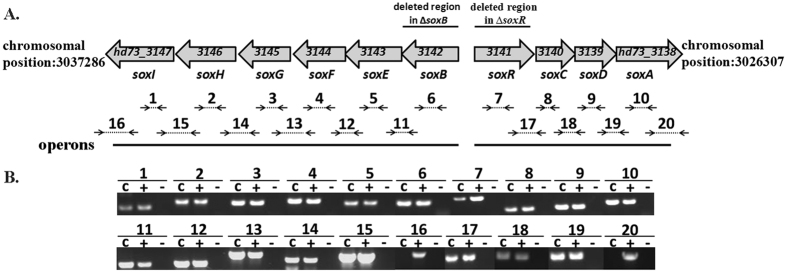
The *sox* locus in Bt HD73 chromosome. Panel A, Map of the *sox* locus in Bt strain HD73. The gray arrows represent ORFs. The positions of fragments that were deleted from the chromosome to disrupt various genes are indicated. Dashed lines with small black arrows annotated with letters correspond to RT-PCR amplicons (see lanes in panel B). The full lines below the ORFs indicate operons. Panel B, RT-PCR analysis of the *sox* locus in Bt strain HD73. The RNA samples were prepared at T_7_ of stationary phase (7 hours after the end of the exponential phase) in SSM. The RT-PCR reactions labeled ‘c’ were performed with 500 ng RNA. The positive controls are labeled ‘+’: PCR with 100 ng genomic DNA. The negative controls are labeled ‘−’: RT-PCR with 500 ng RNA with heat-inactivated reverse transcriptase. The letters refer to the positions of the RT-PCR products, as represented in Fig. 1A.

The nucleotide sequence of the *sox* locus (10,979 bp) of Bt HD73 is comprised of ten open reading frames (ORFs) and encodes ten proteins, which have been annotated as amino acid carrier protein (*soxI*, HD73_3147), aldehyde dehydrogenase (*soxH*, HD73_3146), dihydrodipicolinate synthase (*soxG*, HD73_3145), proline racemase (*soxE*, HD73_3143), sarcosine oxidase, β subunit (*soxB*, HD73_3142), Sigma^54^-dependent transcriptional activator (*soxR*, HD73_3141), hypothetical protein (*soxC/D/F*, HD73_3140/HD73_3139/HD73_3144), and sarcosine oxidase, α subunit (*soxA*, HD73_3138) (Fig. 1A). Identity of Bt Sox proteins to already known function proteins in other bacteria was described in Table S1. The transcriptional units in the *sox* locus were determined by reverse transcription (RT)-PCR. Products were detected for the ten ORFs in this cluster (Fig. 1B, amplicons 1–10). The products between neighboring genes in both the *soxB* and *soxR* orientations were amplified (Fig. 1B, amplicons 11–15 and 17–19); however, no positive signals were detected for amplicons either upstream of *soxI* or downstream of *soxA* (Fig. 1B, amplicons 16 and 20). These results suggest that the *sox* locus is composed of two opposite transcriptional units, *soxB*/*E*/*F*/*G*/*H/I* and *soxR*/*C*/*D*/A.

**Figure 2:**
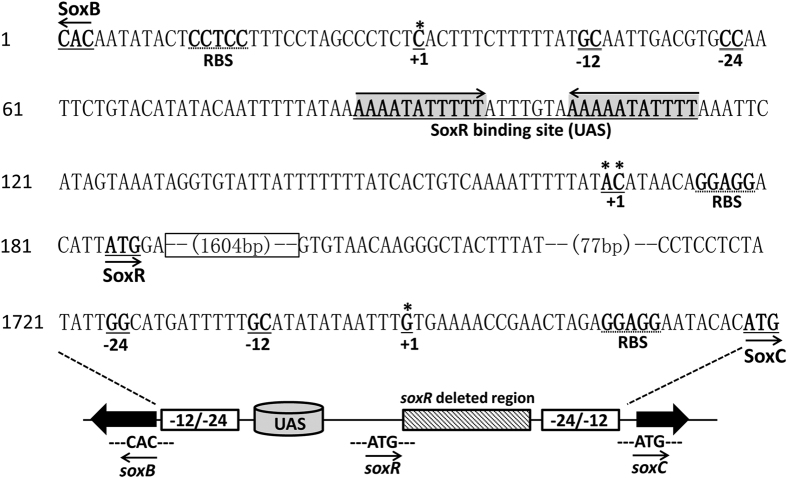
Nucleotide sequence of the intergenic region between the *soxB* and *soxC* genes. The single solid underlined regions with asterisks represent transcriptional start codons. The putative ribosome-binding-site (RBS) is indicated by the single dashed underlined. The -12 and -24 sequences are double-underlined. Transcriptional start sites (TSSs) of the *soxB*, *soxR*, and *soxC* genes are indicated numerically from the TSS (+1) and marked bold characters. The SoxR binding site maps 54 bp upstream of the TSS of *soxB*. A 11-bp repeat region (underlined, gray and arrow) maps 54 bp upstream of the *soxB* TSS. The sequence in the frame represents the *soxR* gene deletion, which is the same fragment deleted in *soxR* mutant.

The sequences GGCACGTCAATTGC and GGCATGATTTTTGC (double underline indicated −12/−24 region of consensus sequence) were located upstream of the *soxB* and *soxC* gene start codons (Fig. 2), respectively, that were similar to the −12/−24 consensus sequence (BYGGCMYRNNNYYGCW) of Sigma^54^-binding sites^27^. The presence of this motif indicates that a third promoter in the *sox* locus may be controlled by the Sigma^54^ factor to direct the transcription of *soxC* and its two downstream genes. DNA microarray data obtained from the HD73 wild-type strain and the *sigL* mutant strain [GEO: GSE48410] revealed that transcription of the six genes of the *soxB* operon is significantly higher in the wild-type strain than in the *sigL* mutant strain^28^, which is consistent with the RT-PCR analysis of the *soxB* operon transcriptional unit (Fig. 1B). Among the four genes of the *soxR* operon, transcription of the *soxC*, *soxD*, and *soxA* genes is higher in the wild-type strain than in the *sigL* mutant. However, transcription of the *soxR* gene did not significantly differ between strains^28^, suggesting that a Sigma^54^-dependent promoter directs the transcription of *soxC*, *soxD*, and *soxA*.

### Determination of the transcriptional start site of *soxB, soxR*, and *soxC*

To determine the TSSs of *soxB, soxR*, and *soxC*, 5′-RACE analysis was performed as described in the materials and methods. According to the sequences of 16 random clones, a C residue located 17 bp upstream from the *soxR* start codon was identified in eight, and an A residue located 18 bp upstream of the start codon in the remaining eight. Thus, two TSSs were located 17 and 18 bp upstream of the ATG start codon of *soxR*. The TSSs of *soxB* and *soxC* were confirmed to be a single 5′-end nucleotide residue G located 28 bp and 29 bp upstream of the start codon according to the sequences of 11 random clones, respectively. Three typical ribosome-binding sites (RBSs) (-GGAGG-) were identified at an appropriate distance upstream of the start codon of *soxB*, *soxR*, and *soxC*. Consistent with the results described above, the sequences upstream of *soxB* and *soxC* proved to be −12/−24 motifs.

### The transcription and regulation of the *soxB* and *soxR* promoters

**Figure 3:**
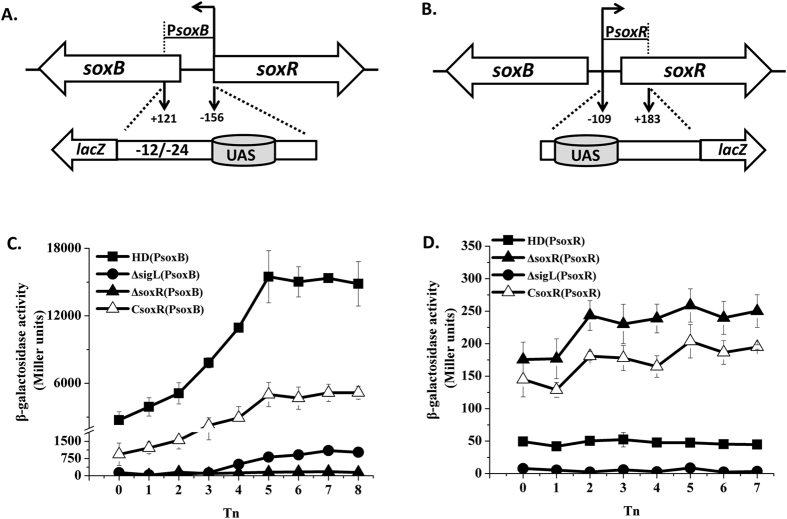
Transcriptional activity of the *soxB* and *soxR* promoters. Panel A, *soxB* promoter region analysis. The indicated promoter region, 156 bp upstream and 121 bp downstream of the TSS, was fused to *lacZ*. Panel B, *soxR* promoter region analysis. The indicated promoter region, 109 bp upstream and 183 bp downstream of the TSS, was fused to *lacZ*. Panel C, β-galactosidase activity of P*soxB*-*lacZ* in wild-type HD73 (■), the *sigL* (●) and *soxR* mutants (▲), and *soxR* complementary strain (△). Panel D, β-galactosidase activity of P*soxR-lacZ* in wild-type HD73 (■), the *soxR* mutant (▲), the *sigL* (●), and *soxR* complementary strain (△). T_0_ is the end of exponential phase and Tn is n hours after T_0_. Each value represents the mean of at least three replicates.

To characterize the transcription mechanism of the *sox* locus, the promoters of *soxB* and *soxR* were fused with *lacZ* (Fig. 3A,B) and the expression of P*soxB* and P*soxR* was assayed in HD73 wild-type, the *sigL* and *soxR* mutants (Fig. 3C,D). The results showed that the β-galactosidase activity of P*soxB* in HD73 wild-type increased from T_0_ to T_5_ and remained high after T_5_. However, it was abolished from T_0_ to T_3_ and significantly reduced after T_3_ in the *∆soxR* and *∆sigL* mutants (Fig. 3C). The activity of P*soxB* recovered in *soxR* complementary strain from T_0_–T_8_, but not reached to wild type level. These results suggest that the transcriptional activity of the *soxB* promoter is dependent on Sigma^54^ and activated by SoxR. The promoter P*soxR* showed lower activity than P*soxB* in HD73 from T_0_ to T_7_, and was lower in HD73 than in the *∆soxR* mutant (Fig. 3D), suggesting transcription of the *soxR* operon is negatively autoregulated. The activity of P*soxR* slightly recovered in *soxR* complementary strain from T_0_–T_7_, but not reached to wild type level. However, the activity of P*soxR* decreased in *∆sigL* mutant compared to that of HD73 wild-type (Fig. 3D), suggesting Sigma^54^ involved in autoregulation of *soxR*.

### Identification of a SoxR-binding site in the *soxB* promoter fragment

**Figure 4:**
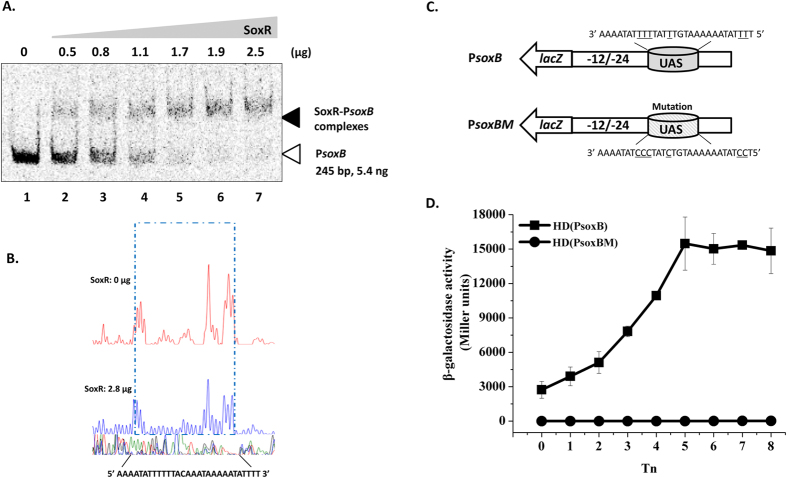
Identification of the SoxR-binding site in the *soxB* promoter. Panel A, Mobility shift assay of the *soxB* promoter fragment (245 bp) after interaction with SoxR. Lane 1, FAM-labeled P*soxB* probe; lanes 2–7, incubation of the probe with increasing concentrations of purified SoxR indicated at the top of the figure. Each lane contained 5.4 ng of probe. Panel B, protection of a 29-bp sequence in the *soxB* promoter by SoxR, as revealed by DNase I footprinting protection assay. The fluorograms correspond to the DNA in the protection reactions (with 0 and 2.8 μg SoxR). Panel C, *soxB* promoter analysis. The indicated promoter regions with the wild-type or mutated SoxR binding site (underlined), P*soxB* and P*soxBM* were fused to *lacZ*. Panel D, β-galactosidase activity assay of the *soxB* promoter with the wild-type SoxR-binding site (■) and mutated SoxR-binding site (●). T_0_ is the end of exponential phase, and Tn is n hours after T_0_. Each value represents the mean of at least three replicates.

To determine whether SoxR binds to the *soxB* promoter, SoxR-His protein was expressed in *E. coli* and purified to near-homogeneity by Ni^2+^-affinity chromatography (Fig. S1). The ability of SoxR to bind to a DNA fragment containing P*soxB* (245 bp) was examined by EMSA. FAM-labeled fragments containing the promoter regions of *soxB* were incubated with different amounts of SoxR and assayed for the formation of protein-DNA complexes. Slower-migrating probe-protein complexes were observed upon incubation with increasing amounts of SoxR (Fig. 4A). Competitive gel shift assays were performed with labeled DNA probes and about 100-fold of the unlabeled DNA targets (specific competitors) respectively. As shown in Fig. S1, 100-fold excess of *soxB* promoter probe could dissociate most of the SoxR from labeled *soxB* promoter probe. Thus, SoxR recognizes and specifically binds to sequences within the *soxB* promoter fragment.

To precisely determine the SoxR-binding site in the *soxB* promoter, DNase I footprinting assays were performed using the same *soxB* promoter fragment used in the EMSA. A fragment (5′-AAAATATTTTTTACAAATAAAAATATTTT-3′) were protected by SoxR binding (Fig. 4B) (corresponding to the shaded gray and underlined sequence in the *soxB* promoter region shown in Fig. 2). Moreover, an 11-bp repeat region mapped 54 bp upstream of the TSS of *soxB* (Fig. 2), with the 11-bp inverted repeat separated by 7 bp (shaded gray in Fig. 2).

To determine whether the proposed sequence is the SoxR binding site *in vivo*, a 278-bp fragment containing the binding site was mutated from the *soxB* promoter and the promoter carrying the mutation was fused to *lacZ* (P*soxBM*) (as described in Methods) (Fig. 4C). The activity was sharply reduced in HD(P*soxBM*) versus the wild-type HD73 carrying the *soxB* promoter fused to *lacZ* (P*soxB-lacZ*) (Fig. 4D). This result suggests that disruption of the proposed SoxR-binding site prevents SoxB expression *in vivo*.

### Identification and regulation of the *soxC* promoter

**Figure 5:**
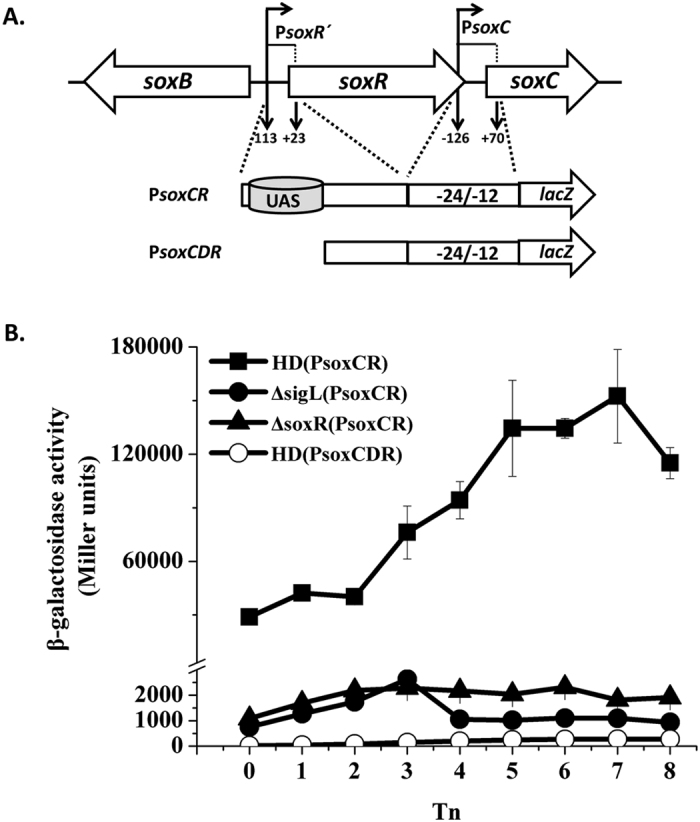
Identification and regulation of the *soxC* promoter. Panel A, *soxC* promoter analysis. The P*soxC* region is located 126 bp upstream and 70 bp downstream of the *soxC* TSS. The P*soxCR* region contains P*soxC* and the fragment located 113 bp upstream and 23 bp downstream of the *soxR* TSS, and contains a SoxR binding site. The P*soxCDR* region contains P*soxCR* and no SoxR binding site. These regions were fused to *lacZ*. Panel B, activity of P*soxCR* site in wild-type HD73 (■) and the *sigL* (●) and *soxR* mutants (▲), and P*soxCDR* promoter without the SoxR-binding site in wild-type HD73 (○). T_0_ is the end of exponential phase, and Tn is n hours after T_0_. Each value represents the mean of at least three replicates.

To identify the promoters of the *soxC/D/A* genes, a putative promoter fragment (P*soxC*) located 126 bp upstream and 70 bp downstream of the TSS of *soxC* was fused with the *lacZ* reporter gene (Fig. 5A). This fusion showed no transcriptional activity (Fig. S2), even though a putative −12/24 motif upstream of *soxC* was identified, suggesting that this fragment did not contain a binding site for SoxR activation of the Sigma^54^-dependent promoter. P*soxCR*, which contains P*soxC* and P*soxR* with the SoxR-binding site, was fused with the *lacZ* gene (Fig. 5A), and showed significantly higher transcriptional activity in comparison to P*soxR* from T_0_ to T_8_ (Fig. 5B). However, the transcriptional activity of P*soxCR* was greatly reduced in *∆soxR* and *∆sigL* mutants (Fig. 5B), suggesting that P*soxCR* contains two promoter regions: a P*soxR* promoter, which directs transcription of the *soxR* operon with low-level activity, and a P*soxC* promoter, controlled by Sigma^54^ and positively regulated by SoxR with high-level activity to direct the transcription of the *soxC*, *soxD*, and *soxA* genes of the *soxR* operon.

To determine whether the SoxR-binding site is necessary for expression from P*soxCR*, a 115-bp fragment containing the SoxR-binding sites was deleted from the P*soxCR* promoter, and the 5′-truncated promoter carrying the deletion was fused to *lacZ* (P*soxCDR-lacZ*) (as described in Methods). The β-galactosidase activity of HD(P*soxCDR*) strain was abolished in comparison to wild-type HD73 carrying the P*soxCR* promoter (Fig. 5B). Thus, the SoxR-binding site is required for Sigma^54^-dependent activity of *soxC* promoter, suggesting that SoxR binds to the same sequence to activate the transcription of both *soxB* and *soxC* genes.

### The *sox* locus is responsible for the utilization of sarcosine

The *soxA* and *soxB* genes in the *sox* locus were annotated as the sarcosine oxidase α and β subunits. Sarcosine oxidase catalyzes the oxidative demethylation of sarcosine to yield glycine and has been implicated in creatine and glycine betaine metabolism in some bacteria^29^,^30^,^31^. To evaluate the metabolic role of this locus, mutants with *soxB* disruptions were constructed. The growth of various strains was tested using sarcosine, proline, creatine, glycine betaine, and glycine, as the sole nitrogen sources in glucose minimal medium. The results demonstrated that Bt HD73 utilizes sarcosine, proline, creatine, and glycine betaine (Table 1). The disruption of *soxR* greatly reduced expression of the *soxR* and *soxB* operons. The doubling time of the *soxR* and *soxB* mutants exceeded 60 h in the presence of sarcosine as the sole nitrogen source, and no effect on proline utilization was observed (Table 1). These results clearly indicated that the *soxR* and *soxB* operons are responsible for sarcosine utilization. All mutants grew in medium containing either creatine or glycine betaine as a nitrogen source.

## Discussion

Transcription of the *sox* locus, which encodes sarcosine oxidase, is regulated in a Sigma^54^-dependent manner in Bt. Transcriptional regulation of the *sox* locus has been studied in only a few bacteria. The sarcosine-responsive transcription factor SouR regulates the *soxBDAG* operon in *Pseudomonas aeruginosa*^32^. Both of the putative promoter regions of *soxA* gene and the reverse-strand *soxR* gene possess −10 and −35 sequences in *Arthrobacter* sp. TE1826^5^,^7^, suggesting that the *sox* locus is regulated by Sigma^70^ in this bacterium. Thus, regulation of the *sox* locus varies in Bt.

In this study, three promoters of two reversed operons were identified in the *sox* locus of Bt. P*soxB* and P*soxCR* were found to be regulated by the Sigma^54^ factor. The Sigma^54^-dependent promoter requires an EBP to trigger Sigma^54^ factor activity^33^,^34^. Sigma^54^-dependent loci typically contain an EBP-encoded gene that is directed by a Sigma^54^-independent promoter and a Sigma^54^-dependent gene or operon. For example, the *levDEFG*^35^, *bkd*^36^, and *acoABCL*^37^ operons in *B. subtilis* are transcribed from Sigma^54^-dependent promoters and are positively regulated by their EBP (LevR, BkdR, and AcoR proteins); these EBP-encoded genes are regulated through a Sigma^54^-independent promoter. Few studies have shown that three promoters drive the transcription at the Sigma^54^-dependent gene locus (Fig. S3). For example, *roc* locus (the *rocABC* and *rocDEF* operons and the *rocG* gene) encode the relevant enzymes of the arginine pathway in *B. subtilis*^23^,^38^,^39^. There are three conserved −12/−24 motifs upstream of the *rocG*, *rocA*, and *rocD* genes, and they are expressed in the same direction that is positively regulated by RocR. The *rocG*-*rocABC* intergenic region acts as both a downstream activating sequence (DAS) and an upstream activating sequence (UAS) for RcoR protein binding^40^. A UAS was identified upstream from the translational start codon of the *rocDEF* operon, which is similar to the *rocABC* operon^23^. The *sox* locus in Bt also has three promoters (Fig. S3), two of them are regulated by the Sigma^54^ factor. The *soxB* and *soxC* genes have conserved −12/−24 motifs, but the promoter of the *soxR* gene is not a Sigma^54^-dependent promoter, but is negatively regulated by SoxR in an unknown manner. In contrast to the *rocABC* operon, the *rocDEF* operon and the *rocG* gene are in the same orientation, and the *soxB* operon in the *sox* locus is located in the opposite direction of the *soxR* operon.

The binding site of SoxR is located far from the −12/−24 motif of *soxC* in Bt. The distance from the first G of the −12 element to the A of the translational start codon of the *soxR* gene is at least 1731 bp. A previous report indicated that the EBP binding sites retain partial activity and activate gene expression, even when located far from its promoter. For example, moving the binding sites for NRI more than 1000 bp does not diminish the ability of NRI to stimulate transcription of *glnAp2* in the *E. coli glnALG* operon^41^. Expression of the *rocG* gene in *Bacillus subtilis* depends on the binding site for RocR, which is located 1.5 kb downstream of its promoter. Furthermore, this activating sequence can be moved as far as 15 kb downstream of the *rocG* promoter and still retain partial activity^39^. In this study, we also demonstrated that the binding site of SoxR retains activity for the *soxC* gene although it is located far from the −12/−24 motif of the *soxC* gene (at least 1.7 kb). We proposed that the EBP binding site contains a large number of A or T tracts that are in phase with the DNA helix pitch, which might cause sequence curvature, thereby facilitating the interaction of SoxR with a Sigma^54^-RNA polymerase, such as RocR^40^. However, the PsoxCRW promoter, which contains the *soxR* promoter, *soxR* gene and *soxC* promoter, had no activity in HD73 or the *soxR* mutant strains (Fig. S2). Expression of the *soxR* gene was negatively autoregulated through the promoter P*soxR*, suggesting that the level of SoxR protein expression fine-tunes this regulation in Bt. Thus, P*soxCRW* may show no promoter activity due to the overexpression of SoxR. It is also a possible reason in that the activity of P*soxB* in a genetically complementary strain with *soxR* was lower than that in wild-type strain (Fig. 3C).

The activity of P*soxR* decreased in *∆sigL* mutant compared to that of HD73 wild-type (Fig. 3D), suggesting Sigma^54^ involved in autoregulation of *soxR*. In *sigL* mutant strain, Sigma^54^ could not interaction with SoxR, and SoxR specific autoregulated its own promoter. Consequently, the activity of P*soxR* decreased in *sigL* mutant. However, *soxR* promoter region has no typical −12/−24 conserved sequence, indicating that Sigma^54^ could not directly control the transcription of *soxR*. The promoters of *soxB*, *soxC* and *soxR* are regulated by SoxR and used the same SoxR binding site UAS, suggesting that SoxR has a precise regulatory mechanism. The transcription of *soxB* and *soxC* are controlled by Sigma^54^ through the interaction with SoxR binding with UAS. In wild type strain HD73, P*soxR* was precisely autoregulated by SoxR interacted with Sigma^54^. However, the activity of P*soxR* decreased by SoxR autoregulation binding with UAS without Sigma^54^ interaction in *sigL* mutant. It indicates that Sigma^54^ positively regulates P*soxR* promoter through SoxR as its interaction protein, and does not play a role as the sigma factor.

In this study, we showed that the *sox* locus of Bt HD73 is essential for sarcosine utilization. The *soxA* and *soxB* genes encode proteins with high sequence similarity to the sarcosine oxidase alpha and beta units^42^,^43^. Disruption of the *soxB* gene abolished sarcosine utilization in Bt. Conserved domain analysis showed that the SoxC and SoxD proteins contain a 2Fe-2S binding domain, with a 2Fe-2S cluster, which appears in amino acid oxidase proteins^44^ as well as sarcosine oxidase. Thus, the functions of the SoxC and SoxD proteins might be similar to that of sarcosine oxidase, although the functions of the proteins encoded by the *soxI/H/G/F/E* genes of the *soxB* operon remain unknown. All of these proteins showed low sequence similarity and shared no conserved domains with the enzymes involved in the metabolism of creatine, glycine betaine, and sarcosine in *Arthrobacter*^4^,^5^ and *Corynebacterium*^9^,^10^.

The orthologs of the *sox* locus of Bt HD73 are conserved in the genome of *B. cereus*^13^,^45^,^46^. These genes share high sequence similarity and a similar organization as the *sox* locus. A similar organization and Sigma^54^-dependent transcription activator of the *sox* locus have been identified in other *Bacillus cereus* species (Fig. S4) and the domains of SoxR from Bt HD73 were conserved in the genomes of these strains. Further analysis of *sox* locus in these strains revealed a series of putative −12/−24 motifs (Fig. S5). All of these suggest Sigma^54^ regulates the expression of sarcosine oxidase in these bacteria.

**Figure 6:**
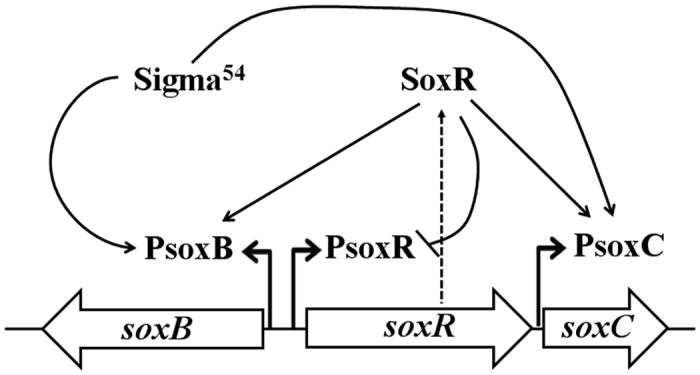
Schematic representation of *sox* locus regulation in Bt strain HD73. The hollow arrows (from left to right) in the middle of the figure indicate the organization of the *sox* locus in Bt HD73. The magnified section of the figure shows the promoters of *soxB*, *soxR*, and *soxC* (which are regulated by SoxR), in which PsoxB, PsoxR, and PsoxC represent the putative promoter regions (marked with angled arrows). The single arrows represent positive regulation, the double arrow between *soxR* and Sigma^54^ represents interdependence, and the block arrow represents negative regulation. The dotted arrows indicate translation.

We propose a transcription model for the *sox* locus in Bt HD73 (Fig. 6). The *sox* locus in Bt has two opposing operons, *soxR* (four genes) and *soxB* (six genes), which contain two Sigma^54^-regulated promoters, P*soxB* and P*soxC.* Sigma^54^ and SoxR regulate the *sox* locus. RNA polymerases containing Sigma^54^ recognize the conserved −12/−24 promoter sequence of the *soxB* and *soxC* genes and generate closed complexes, while SoxR stimulates the isomerization of the closed complexes to open complexes, thus activating the transcription of the *soxB* operon and the *soxC/D/A* genes. The sarcosine oxidase encoded by this locus catalyzes sarcosine to generate glycine. The expression of *soxR*, which is controlled by P*soxR*, is negatively autoregulated.

## Methods

### Bacterial strains, plasmids, and growth conditions

The bacterial strains and plasmids used in this study are listed in Table S2. *Bacillus thuringiensis* subsp. *kurstaki* strain HD73 from the Centre OILB (Institut Pasteur, France) that is deposited in Bacillus Genetic Stock Center (BGSCID. 4D4) was used throughout the study (accession numbers CP004069)^26^. *E. coli* strain TG1 was used as the host for cloning experiments. The Dam^−^/Dcm^−^ *E. coli* ET12567 strain (laboratory stock) was used to generate unmethylated DNA for the electrotransformation assay. Bt strains were transformed by electroporation, as described previously^47^. *E. coli* were cultured in Luria-Bertani (LB) medium, with 220 rpm shaking, at 37 °C. Bt was grown in LB medium, Schaeffer’s sporulation medium (SSM)^48^, or glucose minimal medium (GMM)^49^ supplemented with 40 mM of a given amino acid as the sole nitrogen source, with vigorous shaking (220 rpm) at 30 °C. The antibiotic concentrations used for bacterial selection were as follows: 100 μg/ml kanamycin and 10 μg/ml erythromycin for Bt, and 100 μg/ml ampicillin for *E. coli*.

### DNA manipulation techniques

PCR was performed using *Taq* and Primestar DNA polymerase (TaKaRa Biotechnology, Dalian, China). Amplified fragments were purified using Axygen purification kits (Silicon Valley, CA, USA). Bt chromosomal DNA was extracted with the Puregene kit (Gentra, Minneapolis, MN, USA). Restriction enzymes and T4 DNA ligase (TaKaRa Biotechnology, Dalian, China) were used according to the manufacturer’s instructions. Oligonucleotide primers (Table S3) were synthesized by Sangon (Shanghai, China). *E. coli* plasmid DNA was extracted using the Axygen Plasmid Extraction Kit. All constructs were confirmed by DNA sequencing (BGI, Beijing, China).

### Total RNA isolation, reverse transcription PCR (RT-PCR)

Total RNA was extracted at stage T_7_ from Bt cells grown in SSM, and the RT-PCR analysis was performed as described^50^ using primers RT-1 to RT-20. The 16S rRNA gene was PCR-amplified in all samples using the 16SrDNA5/16SrDNA3 primers to verify the absence of DNA contamination.

### 5′-RACE analysis

The extraction and purification of total RNA were performed as described above. cDNA synthesis and transcriptional start sites (TSSs) of *soxB*, *soxR* and *soxC* were determined using the SMARTer^TM^ RACE cDNA Amplification Kit (Clontech, Mountain View, CA) according to manufacturer instructions. Gene-specific primers and universal primer mix were used to amplify the 5′ end of *soxB*, *soxR*, and *soxC* mRNA.

### Construction of *soxB* and *soxR* promoters with *lacZ* gene fusion

Fragments of the *soxB* (278 bp) and *soxR* (128 bp) promoters were PCR-amplified from strain HD73 DNA using primers PsoxB-F/PsoxB-R and PsoxR-F/PsoxR-R, respectively. The *Pst*I-*Bam*HI restriction fragments were then ligated into pHT304-18Z, which contains a promoterless *lacZ* gene^51^. The recombinant pHT-PsoxB and pHT-PsoxR plasmids were introduced into Bt HD73, Δ*sigL*, and Δ*soxR* mutant strains, to yield HD73(PsoxB), Δ*sigL*(PsoxB), Δ*soxR*(PsoxB), HD73(PsoxR), Δ*sigL*(PsoxR), and Δ*soxR*(PsoxR), which were selected by resistance to erythromycin and verified by PCR.

### Construction of a P*soxC*-*lacZ* fusion

Two DNA fragments of the *soxC* promoter were fused with *lacZ*. A 197-bp fragment located between −126 bp and +70 bp was PCR-amplified from strain HD73 with primers PsoxC-F/PsoxCR-R. Another 334-bp fragment was amplified from the Δ*soxR* mutant strain with primers PsoxCR-F/PsoxCR-R. This fragment contained the putative SoxR binding site. The two *Pst*I-*Bam*HI restriction fragments were then ligated into the pHT304-18Z vector. The recombinant plasmids pHT-PsoxC and pHT-PsoxCR were introduced into the Bt HD73, Δ*sigL*, and Δ*soxR* mutant strains to yield HD73(PsoxC), Δ*sigL*(PsoxC), Δ*soxR*(PsoxC), HD73(PsoxCR), Δ*sigL*(PsoxCR), and Δ*soxR*(PsoxCR). Transformants were selected by resistance to erythromycin and verified by PCR.

### Construction of the P*soxC-lacZ* fusion bearing deletion of the SoxR-binding site

A fragment containing the 5′-truncated *soxC* promoters with the SoxR-binding site deleted was fused to *lacZ*. The construct was obtained as follows: The 219-bp fragment was PCR-amplified from pHT-PsoxCR with primers PsoxCDR-F/PsoxCR-R. The fragment did not contain the SoxR-binding site. The recombinant plasmid pHT-PsoxCDR was introduced into Bt HD73 to produce HD73(PsoxCDR), which were selected by resistance to erythromycin and verified by PCR. The β-galactosidase activity was determined as previously described^52^ and expressed as Miller units. Reported values represent averages from at least three independent assays.

### Expression and purification of SoxR

The expression plasmid pETsoxR containing *soxR* from Bt strain HD73 was constructed by amplifying *soxR* with primers SoxR-F and SoxR-R and cloning into *Bam*HI/*Sal*I-digested pET21b. pETsoxR was transferred into *E. coli* BL21(DE3) and the transformants were grown to the exponential phase in LB medium supplemented with ampicillin at 37°C. The expression and purification of SoxR-His protein was performed as previously described^53^.

### Gel mobility shift assays and DNase I footprinting assays

DNA fragments were PCR-amplified from HD73 genomic DNA using specific primers labeled with a 5′-end 6-FAM modification and confirmed by DNA sequencing. Electrophoresis mobility shift assays (EMSA) were performed as described^54^ to analyze the binding of purified SoxR to P*soxB* DNA containing the putative SoxR binding site. The specificity of the shift was confirmed using poly (dI:dC), and GST protein, bovine serum albumin (BSA), and as negative controls. DNase I footprinting assays were performed based on a fluorescence labeling procedure^53^.

### Construction of the P*soxB-lacZ* fusion bearing mutation of SoxR-binding site

A fragment containing the *soxB* promoter with the SoxR-binding site mutated was cloned in fusion with the *lacZ* gene. The construct was obtained as follows: The fragment (278 bp) with the SoxR-binding site was mutated (A to G, the mutation site was indicated in Fig. 4) by gene synthesis (GENEWIZ, Suzhou, China) and ligated into pHT304-18Z. The recombinant plasmids named pHT-P*soxBM* was introduced into Bt strain HD73, yielding the transformant strains HD(P*soxBM*).

### Construction of the *soxR* and *soxB* mutants

To construct the *soxR* deletion mutant, DNA fragments corresponding to the upstream and downstream regions of *soxR* were first PCR-amplified from genomic Bt HD73 DNA with the *soxR*-a/*soxR*-d and *soxR*-b/*soxR*-c primers. The amplified fragments were then fused via overlapping PCR using the *soxR*-a/*soxR*-b primers. The resultant 1257-bp fragment was then digested with *Bam*HI and *Eco*RI and ligated into pMAD. The recombinant pMAD∆soxR plasmid was electroporated into host strains, and erythromycin-sensitive transformants were selected. Transformants were verified by culturing at 39 °C–41 °C. Colonies lacking erythromycin resistance were selected, and one mutant strain, Δ*soxR*, was verified by PCR.

The upstream and downstream regions (fragments A and B, respectively) of the *soxB* gene were PCR-amplified from Bt with primers soxB-a/soxB-d and soxB-c/soxB-b. The kanamycin resistance cassette (*kan*) was PCR-amplified from pDG780 with primers soxB-kmF/soxB-kmR. Fragment A and *kan* were ligated by overlapping PCR with primers soxB-a and soxB-kmR. The amplification product was then integrated with fragment B in a second round of overlapping PCR using the soxB-a and soxB-b primers. The resultant PCR products were digested, purified, and ligated with pMAD to yield pMADΔsoxB, which was used to transform the host strain by electroporation, followed by selection of erythromycin-sensitive transformants. Transformants were verified by culturing at 39 °C–41 °C. Colonies with kanamycin resistance but lacking erythromycin resistance were selected, and one mutant strain, Δ*soxB*, was verified by PCR.

### Complementation of the *soxR* mutant

A DNA fragment containing *soxR* and the *soxR* promoter was amplified with CsoxR-1 and CsoxR-2 primers (Table S3) using Bt strain HD73 DNA as template. The PCR product (2,268 bp) was digested with *Pst*I and *Xba*I and ligated into plasmid pHT1618^55^. The resulting plasmid (pHT1618-soxR) was amplified in *E. coli* and introduced into the Bt mutant strain Δ*soxR*(P*soxB*-*lacZ*), and the new strain named CsoxR(PsoxB). This plasmid complements the *soxR* mutant strain and allows evaluation of the expression of the *soxB* promoter-*lacZ* fusion.

## Additional Information

**How to cite this article**: Peng, Q. *et al*. *Sox* transcription in sarcosine utilization is controlled by Sigma^54^ and SoxR in *Bacillus thuringiensis* HD73. *Sci. Rep.* **6**, 29141; doi: 10.1038/srep29141 (2016).

## Supplementary Material

Supplementary Information

## Figures and Tables

**Table 1:** Doubling time of HD73 wild-type and mutant strains grown in minimal medium containing various nitrogen sources.

Nitrogen source	Doubling time (hour)		
	HD73	*ΔsoxR*	*ΔsoxB*
Sarcosine	21.55 ± 2.17	>60	>60
Proline	21.12 ± 2.08	24.24 ± 2.98	24.08 ± 2.23
Creatine	27.02 ± 2.70	29.30 ± 7.85	26.62 ± 6.65
Glycine betaine	17.14 ± 2.91	18.69 ± 1.17	16.85 ± 2.39
Glycine	>60	>60	>60
