# Supplementary material for: Sox transcription in sarcosine utilization is controlled by Sigma54 and SoxR in Bacillus thuringiensis HD73
Source: Sci Rep. 2016 Jul 12;6:29141. doi: 10.1038/srep29141 (PMC4941409; doi:10.1038/srep29141)
Supplement: Supplementary Information [file srep29141-s1.pdf]

***Sox* transcription in sarcosine utilization is controlled by Sigma^{54} and SoxR in *Bacillus thuringiensis* HD73**

Qi Peng[1], Chunxia Liu[1], Bo Wang[1, 2], Min Yang[1], Jianbo Wu[1], Jie Zhang[1], Fuping Song[1]*

[1]State Key Laboratory for Biology of Plant Diseases and Insect Pests, Institute of Plant Protection, Chinese Academy of Agricultural Sciences, Beijing, China

[2]College of Life Sciences, Northeast Agriculture University, Harbin, China

[*]Address correspondence to Fuping Song,

No.2 West YuanMingYuan Road, Beijing, 100193, China

Phone number: +86 10 62896634

Fax number: +86 10 62812642

E-mail: fpsong@ippcaas.cn

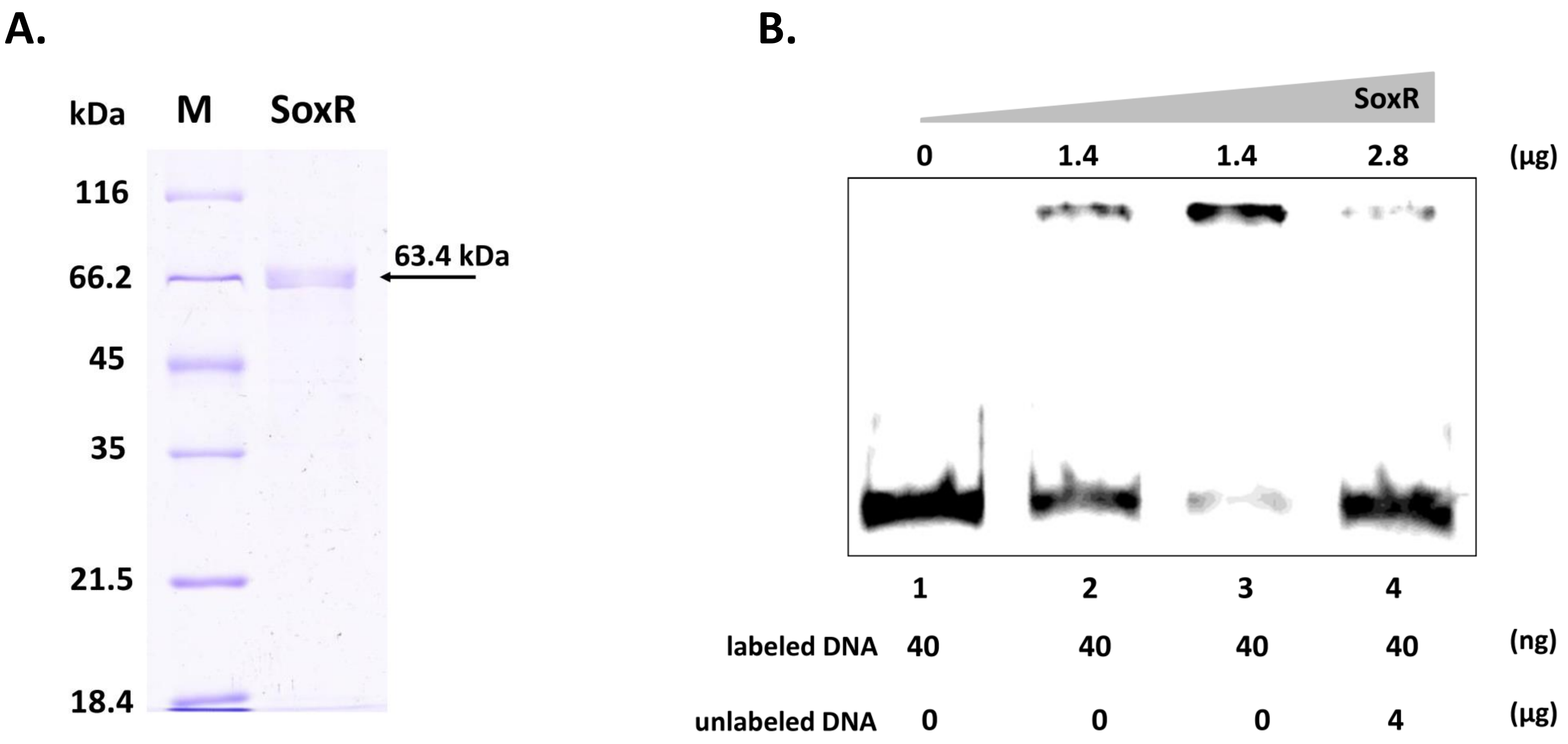

Figure S1. Panel A, SDS-PAGE analysis of SoxR (63.4 kDa) expressed in *E. coli* (pET21b-soxR) and purified by nickel affinity column chromatography. M, protein marker. Panel B, Competitive gel shift assays. Lane 1, FAM-labeled P*soxB* probe; lanes 2-3, incubation of the probe with increasing concentrations of purified SoxR indicated at the top of the figure; lane 4, incubation of SoxR with FAM-labeled P*soxB* probe and unlabeled P*soxB* probe, respectively.

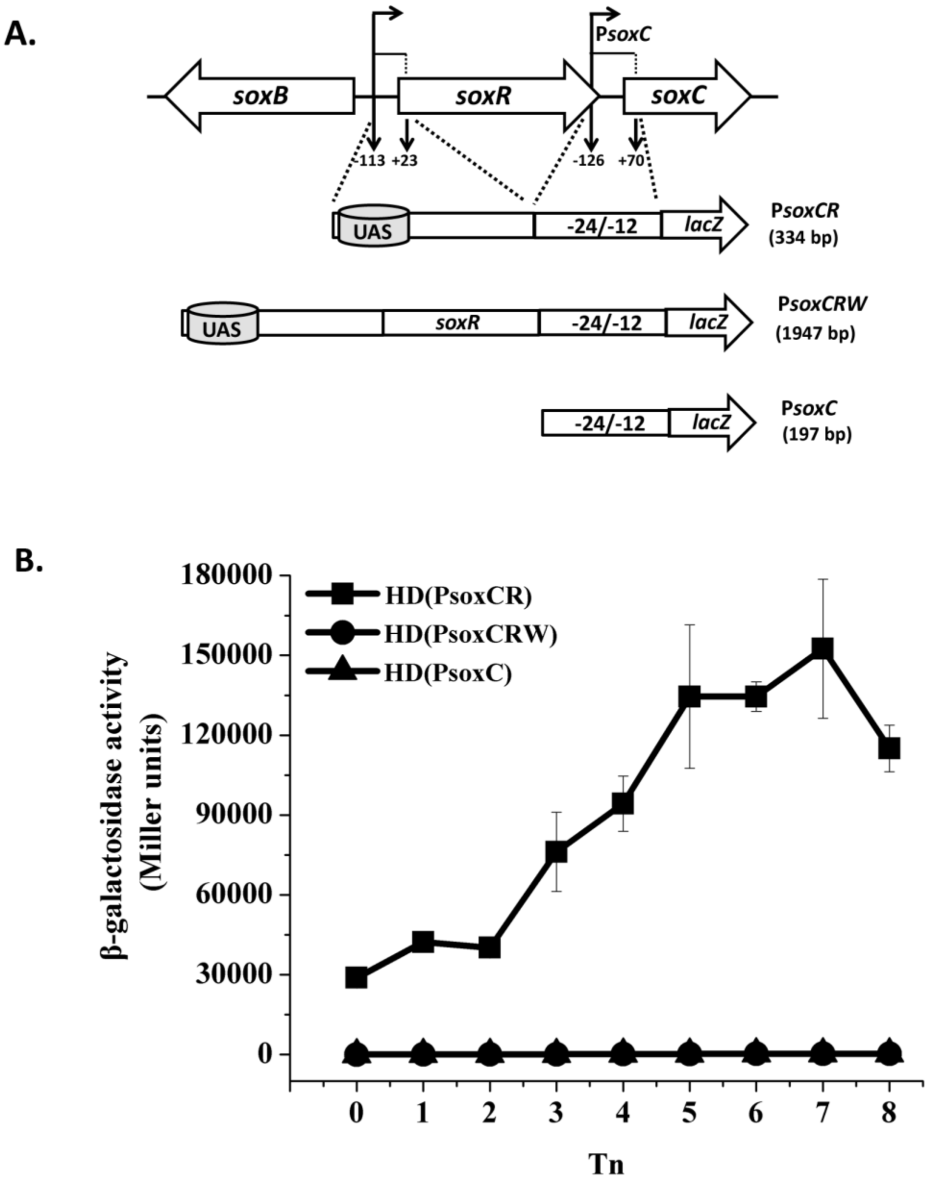

FIGURE S2.

Panel A, *soxC* promoter analysis. The P*soxC* region is located 126 bp upstream and 70 bp downstream of the *soxC* TSS. The P*soxCR* region contains P*soxC* and the fragment located 113 bp upstream and 23 bp downstream of the *soxR* TSS, and contains a SoxR binding site. The P*soxCRW* region contains P*soxCR* and *soxR* gene. These regions were fused to *lacZ*. Panel B, activity of P*soxCR* (■), P*soxCRW* (●) and P*soxC* (▲) in wild-type HD73. T_0 is the end of exponential phase, and Tn is n hours after T_0. Each value represents the mean of at least three replicates.

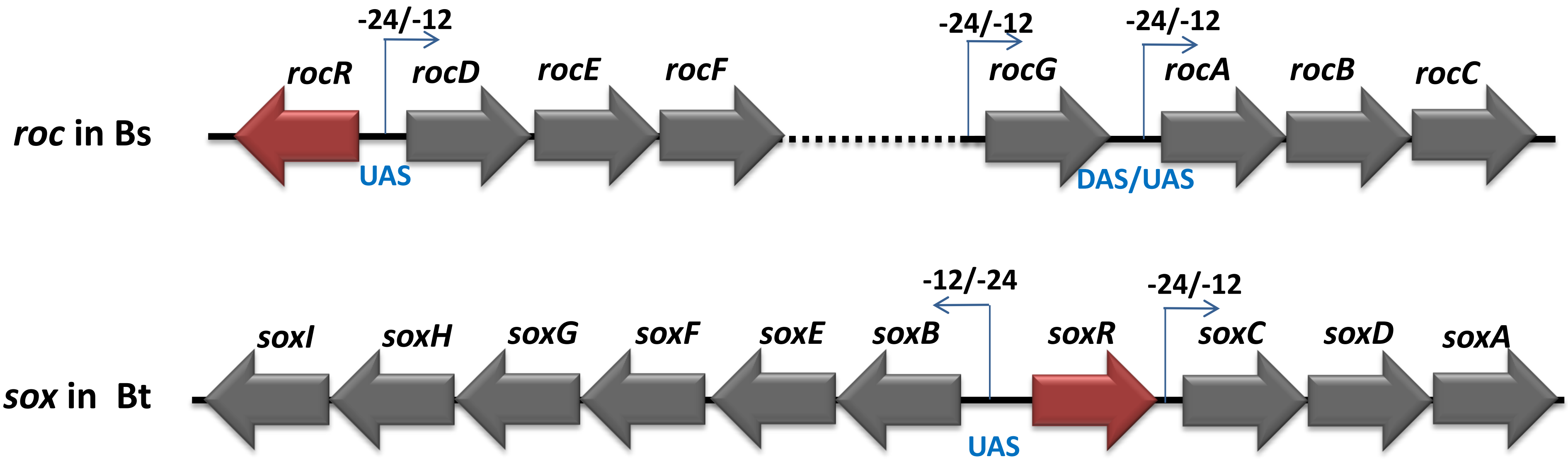

Figure S3. Comparison of the genetic organization and the regulation of *sox* locus in *B. thuringiensis* and *roc* locus in *B. subtilis*.

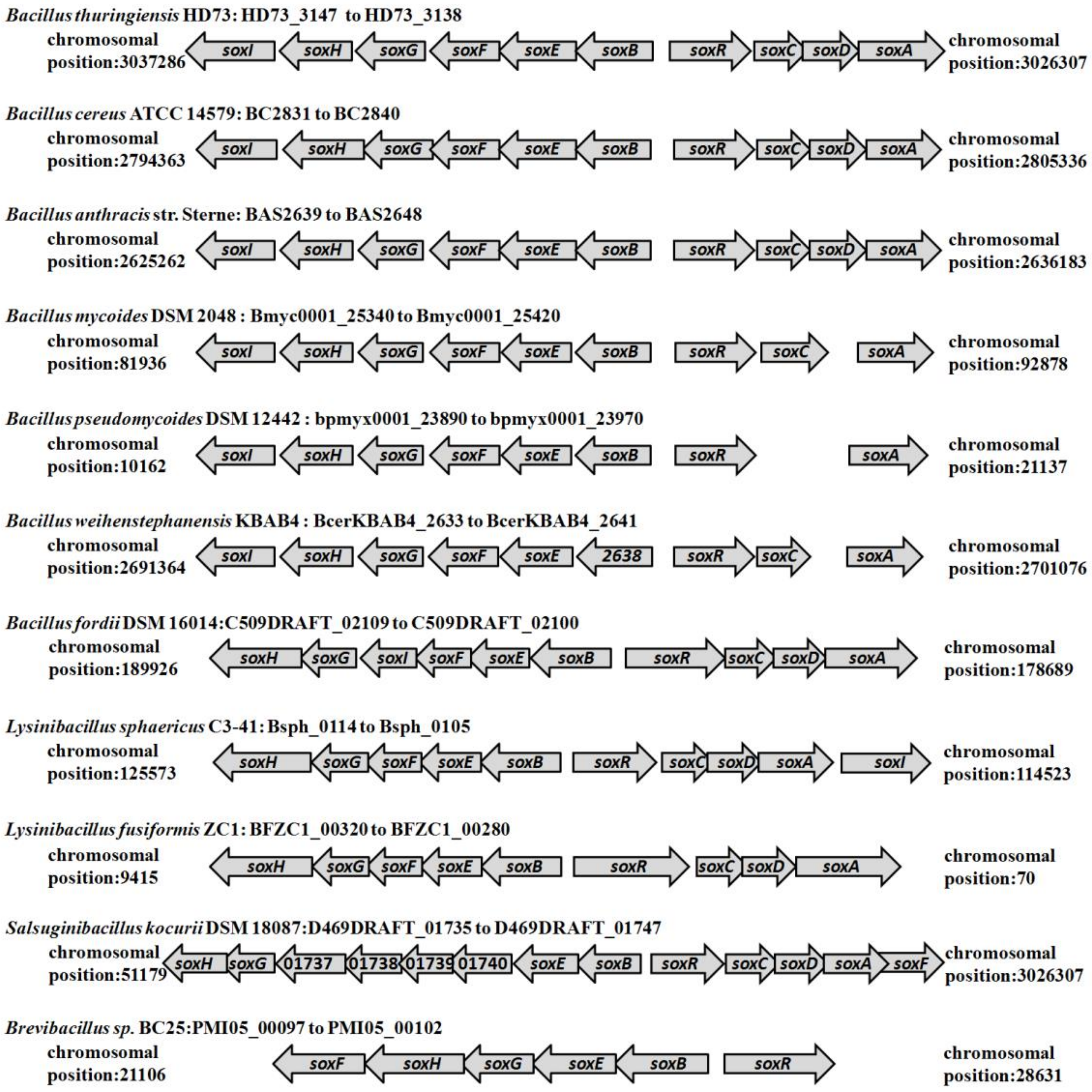

Figure S4. The structure of the *sox* locus in different *Bacillus* species. The corresponding species and the ORFs of each locus are indicated above their structure.

	-24 -12	
**Consensus :**	TGGCACNNNNNTTGCW	
***Bacillus thuringiensis* HD73:**		
	ATTGGCACGTCAATTGCATA	(upstream of *soxB*)
	ATTGGCATGATTTTTGCATA	(upstream of *soxC*)
***Bacillus cereus* ATCC 14579:**		
	GATGGGATTAGTCTTGCAGC	(upstream of *soxE*)
	ATTGGCACGTCAATTGCATA	(upstream of *soxB*)
	ATTGGCATGATTTTTGCTTA	(upstream of *soxC*)
***Bacillus anthracis* str. Sterne:**		
	GTTGGCACGTCAATTGCATA	(upstream of *soxB*)
	ATTGGCATATTTTTTGCATT	(upstream of *soxC*)
***Bacillus mycoides* DSM 2048:**		
	GTTGGCACACCAATTGCATA	(upstream of *soxB*)
	ATTGGCATAGTTTTTGCATA	(upstream of *soxC*)
***Bacillus pseudomycoides* DSM 12442:**		
	ATTGGCTGTTCTATTGCTTA	(upstream of *soxB*)
	GTTGGCATACCAATTGCATA	(upstream of *soxB*)
	ATTGGCATACCTTTTGCATT	(upstream of *soxA*)
***Bacillus weihenstephanensis* KBAB4:**		
	GTTGGCACACCAATTGCATA	(upstream of *soxB*)
	ATTGGCATAGTTTTTGCATA	(upstream of *soxC*)
***Bacillus fordii* DSM 16014 :**		
	TCTGGTATATTTCTTGCATT	(upstream of *soxC*)
	AATGGCACAATCCTTGCAAA	(upstream of *soxB*)
***Lysinibacillus sphaericus* C3-41:**		
	GTTGGCATGTTTTTTGCATC	(upstream of *soxB*)
	GCTGGCTACGAAGTTGCATT	(upstream of *soxG*)
	TATGGCATAAAACATGCAAT	(upstream of *soxI*)
	TTTGGCATAGTTTTTGCATT	(upstream of *soxC*)
	GGGGGTACTTACCTTGCATC	(upstream of *soxR*)
***Lysinibacillus fusiformis* ZC1:**		
	TCTGGCATAGTTTTTGCAGT	(upstream of *soxC*)
	ATTGGCATAGTTTTTGCACA	(upstream of *soxB*)
	GTTGGCTACGAAATTGCATT	(upstream of *soxG*)
***Salsuginibacillus kocurii* DSM 18087:**		
	CTTGGCATGCATATTGCATT	(upstream of *soxG*)
	AATGGCACAATAATTGCATT	(upstream of *soxB*)
	TTTGGCATTGAACTTGCATT	(upstream of *soxC*)
***Brevibacillus* sp. BC25:**		
	ATTGGCATGGCTTTTGCAAA	(upstream of *soxB*)

Figure S5. The promoter region of the *sox* locus which is regulated by Sigma^{54}-dependent transcriptional activator in different *Bacillus* species. Consensus sequences of Sigma^{54}-dependent promoters are presented. The underlined regions represent imperfect -12/-24 consensus sequence.

Table S1. Identity of Bt Sox proteins to similarity function proteins in other bacteria

Genes in Bt	Annotations	Identity	Organism(s)	Reference
HD73_3138 (*soxA*)	sarcosine oxidase, α subunit	25%	SoxA, *Pseudomonas aeruginosa*	1,2
HD73_3139 (*soxD*)	hypothetical protein	29%	SoxA, *Pseudomonas aeruginosa*	1,2
HD73_3140 (*soxC*)	hypothetical protein	-	-	-
HD73_3141 (*soxR*)	Sigma[54]-dependent transcriptional activator	10%	SouR, *Pseudomonas aeruginosa*	2
HD73_3142 (*soxB*)	sarcosine oxidase, β subunit	21%	SoxB, *Pseudomonas aeruginosa*	1,2
HD73_3143 (*soxE*)	proline racemase	45%	PrdF, *Clostridium difficile*	3
HD73_3144 (*soxF*)	hypothetical protein	19%	PrdF, *Clostridium difficile*	3
HD73_3145 (*soxG*)	dihydrodipicolinate synthase	24%	DapA, *Bacteroides thetaiotaomicron*	4
HD73_3146 (*soxH*)	aldehyde dehydrogenase	48%	*Pseudomonas sp*. strain	5
HD73_3147 (*soxI*)	amino acid carrier protein	95%	AlsT, *Bacillus subtilis*	6

- Similarity function protein was not found.

Table S2. Strains and plasmids

Strain or plasmid	Relevant genotype and characteristics[a]	Reference or source
**Strains**		
HD73	Bt subsp. *Kurstaki* carrying the *cry1Ac* gene	Institut Pasteur, France
HD(ΔsigL)	Bt HD73 *sigL* gene mutant	7
HD(ΔsoxR)	Bt HD73 *soxR* gene mutant	This study
HD(PsoxR)	Bt HD73 carrying pHT-PsoxR plasmid; Em^R	This study
ΔsigL(PsoxR)	HD(ΔsigL) carrying pHT-PsoxR plasmid; Em^R	This study
ΔsoxR(PsoxR)	HD(ΔsoxR) carrying pHT-PsoxR plasmid; Em^R	This study
CsoxR(PsoxR)	HD(Δ*soxR*) carrying pHT-PsoxR and pHT1618-soxR plasmid; Em^R and Tet^R	This study
HD(PsoxB)	Bt HD73 carrying pHT-PsoxB plasmid; Em^R	This study
ΔsigL(PsoxB)	HD(ΔsigL) carrying pHT-PsoxB plasmid; Em^R	This study
ΔsoxR(PsoxB)	HD(ΔsoxR) carrying pHT-PsoxB plasmid; Em^R	This study
HD(PsoxBDR)	Bt HD73 carrying pHT-PsoxBDR plasmid; Em^R	This study
HD(PsoxC)	Bt HD73 carrying pHT-PsoxC plasmid; Em^R	This study
HD(PsoxCR)	Bt HD73 carrying pHT-PsoxCR plasmid; Em^R	This study
ΔsigL(PsoxCR)	HD(ΔsigL) carrying pHT-PsoxCR plasmid; Em^R	This study
ΔsoxR(PsoxCR)	HD(ΔsoxR) carrying pHT-PsoxCR plasmid; Em^R	This study
HD(PsoxCDR)	Bt HD73 carrying pHT-PsoxCDR plasmid; Em^R	This study
HDΔsoxB	Bt HD73 *soxB* gene mutant	This study
CsoxR(PsoxB)	HD(Δ*soxR*) carrying pHT-PsoxB and pHT1618-soxR plasmid; Em^R and Tet^R	This study
E. coli TG1	Δ(*lac-proAB*) *supE thi hsd*-5 (*F' traD36 proA*$^+$ *proB*$^+$ *lacI*^{q} *lacZ*ΔM15), general purpose cloning host	8
E. coli ET12567	*F*$^-$ *dam-13*::Tn*9 dcm-6 hsdM hsdR recF143 zjj-202*::Tn*10 galK2 galT22 ara14 pacY1 xyl-5 leuB6 thi-1*, for generation	8

	of unmethylated DNA	
E.coli BL21(DE3)	*E.coli B, F^-, dcm, ompT, hsdS(rB-mB-), gal, λ(DE3)*	Novagen
BL (pET-*soxR*)	BL21(DE3) with pET-*soxR* plasmid	This study
**Plasmids**		
pHT304-18Z	Promoterless *lacZ* vector, Em^R, Ap^R	9
pET21b	Expressional vector, Ap^R, 5.4 kb	Novagen
pMAD	Ap^R, Em^R shuttle vector, thermosensitive origin of replication	10
pHT1618	*E. coli*-*Bt* shuttle, Ap^R, Tet^R	11
pET-*soxR*	pET-21b containing *soxR* gene, Ap^R	This study
pHT-P*soxR*	pHT304-18Z carrying promoter upstream from *soxR*	This study
pHT-P*soxB*	pHT304-18Z carrying promoter upstream from *soxB*	This study
pHT-P*soxBDR*	pHT304-18Z carrying promoter upstream from *soxB* without SoxR binding site	This study
pHT-P*soxC*	pHT304-18Z carrying upstream from *soxC*	This study
pHT-P*soxCR*	pHT304-18Z carrying promoter upstream from *soxR* and *soxC*	This study
pHT-P*soxCDR*	pHT304-18Z carrying promoter upstream from *soxR* and *soxC* without SoxR binding site	This study
pMADΔsoxR	pMAD with *soxR* deletion fragment	This study
pMADΔsoxB	pMAD with *soxB* deletion fragment	This study
pHT1618-soxR	pHT1618 containing *soxR* promter and *soxR* gene, Ap^R	This study

Table S3 Sequences of the oligonucleotide primers used in this study

Primer name	Sequence (5′’→3′)[a]
soxR-a	CGGGATCCACCGCCTAATAAATCATCAGCAAA
soxR-b	CGGAATTCTATTTACTGTCATGCGGCATTCGG
soxR-c	GGAGGACATTATGGAGTGTAACAAGGGCTACT
soxR-d	TAGCCCTTGTTACACTCCATAATGTCCTCCTG
soxB-a	TCCCCCGGGTGGTACTAATTCTAGATCTACTGA
soxB-b	CGCGGATCCCGCATTCTTTACTATCAATTACTG
soxB-c	GCCTACGAGGAATTTAACGTCGCAGTGCCT
soxB-d	CTCAAATGGTTCGCTGGGGTGTTAAACGGA
soxB-kmF	TCCGTTTAACACCCCAGCGAACCATTTGAG
soxB-kmR	AGGCACTGCGACGTTAAATTCCTCGTAGGC
soxR NGSP	CGAGGCTGATGGAACTGAGCGACTATG
soxR GSP	TAATACGAATAAATCGGGGCGAGGCTG
soxB NGSP	GCGCAGCTTCCATCTCTTCGTCTGAC
soxB GSP	CTTGTTGCCGATTTACCCATTGCTGCG
PsoxB-F	AACTGCAGCCATAATGTCCTCCTGTTATG
PsoxB-R	CGGGATCCGATTGTTACGTCTCTTCCGTA
PsoxR-F	AACTGCAGCCAATTCTGTACATATACA
PsoxR-R	CGGGATCCAGTGAATGAGTCATCTAC
PsoxC-F	AACTGCAGGGGCTACTTTATATAACCG
PsoxCDR-F	AACTGCAGCATAACAGGAGGACATTATGG
PsoxCR-F	AACTGCAGCGTGCCAATTCTGTACATATAC
PsoxCR-R	CGGGATCCTCTTCACAACGACAAACGAT
SoxR-F	CGGGATCCGATGGAATTCTCATTTCCGAC
SoxR-R	ACGCGTCGACTTGAAGTCCA AGTTTCTTTAAAC
PsoxB-EF	ACGCTTTCCAAAAATTCTTG
PsoxB-ER	AAAACGTCGCAGTGCCTCAC
16SrDNA5	ATCTTCCGCAATGGACGAAAGTC
16SrDNA3	GGTCTTGCAGCTCTTTGTACCGT
RT-1R	TTGTACAATGGCGGTTTTCCCTTCAG
RT-1F	ATGTTTGGTGGAATTGTTGGTGCAGC
RT-2R	TAGATACGAGTGGACCCATTTCCGTT
RT-2F	CTGAAGCAGGGGCTGCAATTGTAAAT
RT-3R	CCGAGGCACCAACTAAATATGATTCC
RT-3F	GCACGGAGCGGATTGTGCATTAATTA
RT-4R	GTATAGTCTGTAGTGTAGCTTCTCCC
RT-4F	TAATCATGAAGGTTCGGTTCCGCTTC
RT-5R	ATACAGCTAGCTTCGCAGATGTTCCT
RT-5F	GTGAAAGACATTGGGACTATAGAGGC
RT-6R	TTTTGTGCCTCTGACAGAAGCGAGAA
RT-6F	TAGGGCACCAGGAAGTATTCTCGTAT
RT-7F	AGCGAAGGCGCCTTTCATTTCGATTA

RT-7R	CCTGGCCAATTGTAATGAAGCAGTTC
RT-8F	GTCGTTGTGAAGAGGTTACATATGGG
RT-8R	CCAAAAGTAACTGCACGTATTGGTGG
RT-9F	CGGTCATCAATACGGAGCATATGAAC
RT-9R	GATATTCGGATGCTGTTTCCCGCTTT
RT-10F	AAGCGCCTTTCGTACTACTTGCTACT
RT-10R	AGAAGGAGCGAGATGTGCTGCATTTA
RT-11R	CATACACCGATTGTATCGTGACCACA
RT-11F	GTTTACGTCCGTGGACAGAAGATCAT
RT-12R	GAAGCGGAACCGAACCTTCATGATTA
RT-12F	AGGAACATCTGCGAAGCTAGCTGTAT
RT-13R	TAATTAATGCACAATCCGCTCCGTGC
RT-13F	GGGAGAAGCTACACTACAGACTATAC
RT-14R	ATAAGATGCGCTCTTTCAGCAGTAGC
RT-14F	GGAATCATATTTAGTTGGTGCCTCGG
RT-15R	AATTGCTGTCGCTACCCCTGCAATAT
RT-15F	TGGACCAGTTGTTGTCGTACTTCCAT
RT-16R	CGTGAACGCATTATATGTCTCAGCGA
RT-16F	TGGACCAGTTGTTGTCGTACTTCCAT
RT-17R	ATCATTATTCTGCATGTGCGACCACC
RT-17F	GAACTGCTTCATTACAATTGGCCAGG
RT-18F	CCACCAATACGTGCAGTTACTTTTGG
RT-18R	AATAAATACCTCGCGGTGTTCCGCTA
RT-19F	TAGCGGAACACCGCGAGGTATTTATT
RT-19R	AGTAGCAAGTAGTACGAAAGGCGCTT
RT-20F	GGAACAGTTGCAGGTCTATCTATTGC
RT-20R	TCATCACTTCGATCAGCTGGGTCATT

[a]Restriction enzyme sites are underscored.

REFERENCES

1 Wargo, M. J., Szwergold, B. S. & Hogan, D. A. Identification of two gene clusters and a transcriptional regulator required for *Pseudomonas aeruginosa* glycine betaine catabolism. *Journal of bacteriology* **190**, 2690-*2699*, doi:10.1128/JB.01393-07 (2008).

2 Willsey, G. G. & Wargo, M. J. Sarcosine Catabolism in *Pseudomonas aeruginosa* Is Transcriptionally Regulated by SouR. *Journal of bacteriology* **198**, 301-310,

doi:10.1128/JB.00739-15 (2016).

3 Wu, X. & Hurdle, J. G. The *Clostridium difficile* proline racemase is not essential for early logarithmic growth and infection. *Canadian journal of microbiology* **60**, 251-254, doi:10.1139/cjm-2013-0903 (2014).

4 Mank, N., Arnette, A., Klapper, V., Offermann, L. & Chruszcz, M. Structure of dihydrodipicolinate synthase from the commensal bacterium *Bacteroides thetaiotaomicron* at 2.1 A resolution. *Acta crystallographica. Section F, Structural biology communications* **71**, 449-454, doi:10.1107/S2053230X15004628 (2015).

5 Adeboye, P. T., Olsson, L. & Bettiga, M. A coniferyl aldehyde dehydrogenase gene from *Pseudomonas sp*. strain HR199 enhances the conversion of coniferyl aldehyde by Saccharomyces cerevisiae. *Bioresource technology* **212**, 11-19, doi:10.1016/j.biortech.2016.04.003 (2016).

6 Rose, M. & Entian, K. D. New genes in the 170 degrees region of the *Bacillus subtilis* genome encode DNA gyrase subunits, a thioredoxin, a xylanase and an amino acid transporter. *Microbiology* **142 (Pt 11)**, 3097-3101, doi:10.1099/13500872-142-11-3097 (1996).

7 Zhu, L. *et al.* Structure and regulation of the *gab* gene cluster, involved in the gamma-aminobutyric acid shunt, are controlled by a sigma54 factor in *Bacillus thuringiensis*. *Journal of bacteriology* **192**, 346-355, doi:10.1128/JB.01038-09 (2010).

8 Song, F. *et al.* A multicomponent sugar phosphate sensor system specifically induced in *Bacillus cereus* during infection of the insect gut. *FASEB journal :*

official publication of the Federation of American Societies for Experimental Biology **26**, 3336-3350, doi:10.1096/fj.11-197681 (2012).

9 Agaisse, H. & Lereclus, D. Structural and functional analysis of the promoter region involved in full expression of the *cryIIIA* toxin gene of *Bacillus thuringiensis*. *Molecular microbiology* **13**, 97-107 (1994).

10 Arnaud, M., Chastanet, A. & Debarbouille, M. New vector for efficient allelic replacement in naturally nontransformable, low-GC-content, gram-positive bacteria. *Applied and environmental microbiology* **70**, 6887-6891, doi:10.1128/AEM.70.11.6887-6891.2004 (2004).

11 Lereclus, D. & Arantes, O. *spbA* locus ensures the segregational stability of pTH1030, a novel type of gram-positive replicon. *Molecular microbiology* **6**, 35-46 (1992).
